# COVID-19 mass testing and sequencing: Experiences from a laboratory in Western Kenya

**DOI:** 10.4102/ajlm.v11i1.1737

**Published:** 2022-07-22

**Authors:** John N. Waitumbi, Esther Omuseni, Josphat Nyataya, Clement Masakhwe, Faith Sigei, Allan Lemtudo, George Awinda, Eric Muthanje, Brian Andika, Rachel Githii, Rehema Liyai, Gathii Kimita, Beth Mutai

**Affiliations:** 1 Kenya Medical Research Institute (KEMRI)/United States Army Medical Research Directorate-Africa, Basic Science Laboratory, Kisumu Field Station, Kisumu, Kenya; 2 Department of Biological Sciences, University of Embu, Embu, Kenya; 3 Department of Molecular Biology and Bioinformatics, Jomo Kenyatta, University of Agriculture and Technology, Juja, Kenya

**Keywords:** COVID-19, coronavirus, SARS-CoV-2, nasal swab, nasopharyngeal swabs, mass testing, genome sequencing

## Abstract

**Background:**

The Basic Science Laboratory (BSL) of the Kenya Medical Research Institute/Walter Reed Project in Kisumu, Kenya addressed mass testing challenges posed by the emergent coronavirus disease 2019 (COVID-19) in an environment of global supply shortages. Before COVID-19, the BSL had adequate resources for disease surveillance and was therefore designated as one of the testing centres for COVID-19.

**Intervention:**

By April 2020, the BSL had developed stringent safety procedures for receiving and mass testing potentially infectious nasal specimens. To accommodate increased demand, BSL personnel worked in units: nucleic acid extraction, polymerase chain reaction, and data and quality assurance checks. The BSL adopted procedures for tracking sample integrity and minimising cross-contamination.

**Lessons learnt:**

Between May 2020 and January 2022, the BSL tested 63 542 samples, of which 5375 (8.59%) were positive for COVID-19; 1034 genomes were generated by whole genome sequencing and deposited in the Global Initiative on Sharing All Influenza Data database to aid global tracking of viral lineages. At the height of the pandemic (August and November 2020, April and August 2021 and January 2022), the BSL was testing more than 500 samples daily, compared to 150 per month prior to COVID-19. An important lesson from the COVID-19 pandemic was the discovery of untapped resilience within BSL personnel that allowed adaptability when the situation demanded. Strict safety procedures and quality management that are often difficult to maintain became routine.

**Recommendations:**

A fundamental lesson to embrace is that there is no ‘one-size-fits-all’ approach and adaptability is the key to success.

## Background

**FIGURE 1:**
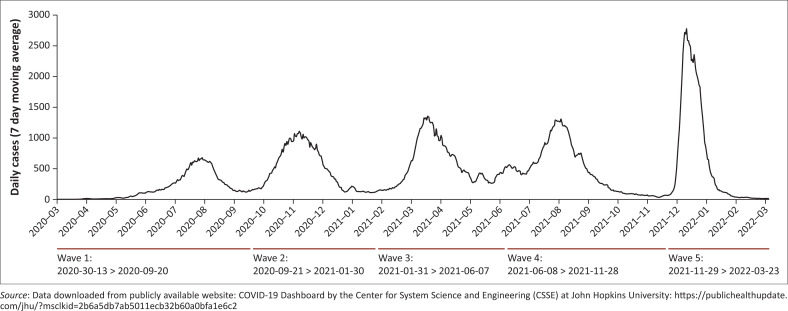
Daily new confirmed COVID-19 cases in Kenya from March 2020 to March 2022.

The first case of coronavirus disease 2019 (COVID-19) was traced to a Kenyan citizen who arrived in Nairobi from the United States on 5th March 2020.^1^ Severe acute respiratory syndrome coronavirus 2 (SARS-CoV-2) was confirmed in the index case on Friday the 13th of March. Kenyans are not known to have *paraskevidekatriaphobia* (“Friday the 13th” superstition), but the coincidence did not escape notice. Through active contact tracing, two contacts who sat next to the index case enroute to Nairobi tested positive 2 days later. Since then, as in the rest of the world, COVID-19 infections have risen dramatically, appearing in waves,^2^ to date at a count of five (Figure 1). Surveillance for the virus and containment measures evolved over time, from original contact tracing, isolation and obligatory testing into mandatory masking, social distancing, lockdown, curfew, social gathering ban, restrictions on local and international flights, and closing of country borders. These strict measures were eventually relaxed and at the time of writing (April 2022), the Kenyan Ministry of Health (MOH) had removed the masking requirement.

**FIGURE 2:**
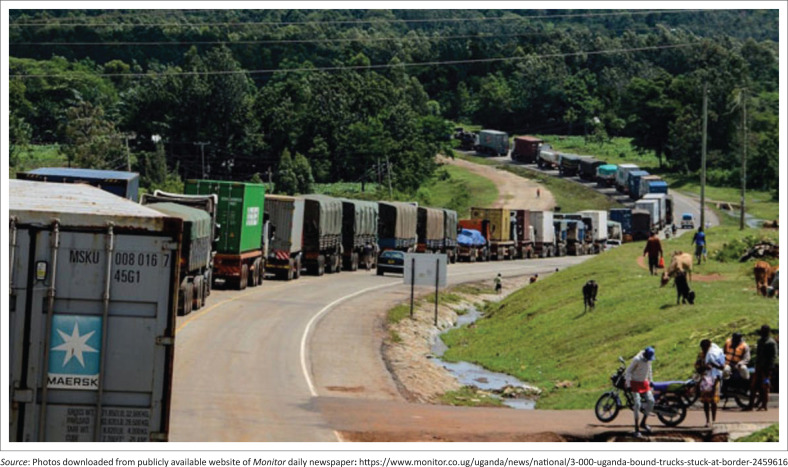
Long queues of trucks at Malaba, Kenya–Uganda border on 29 April 2020 as drivers waited to be certified free of COVID-19.

By early May 2020, it was clear that long-distance truck drivers were transporting the virus across the borders.^3^ Mandatory mass testing and certification for absence of the SARS-CoV-2 became a requirement for all cross-border truck drivers. Testing could not cope with the demand and long queues of trucks held at the border crossing points were common (Figure 2).

Prior to COVID-19, the Kenya Medical Research Institute (KEMRI)/Walter Reed Project, Kisumu Field Station laboratory conducted surveillance for pathogens associated with acute febrile illness and outbreak response, and provided basic science support for clinical trials in western Kenya. The Kenyan government, through the MOH, responded to the COVID-19 pandemic by designating multiple laboratories, including the Basic Science Laboratory (BSL) of the Kenya Medical Research Institute/Walter Reed Project in Kisumu as COVID-19 testing centres that would provide public health support in combatting the pandemic. This report covers May 2020 to January 2022 and aims to document how the BSL rose to the challenges of SARS-CoV-2 diagnostics dictated by increased testing demand amidst global supply shortages.

## Description of the intervention

### Ethical considerations

The work reported here was conducted as part of public health support to combat the COVID-19 outbreak in Kenya. A human subject research protocol review was therefore not required.

### Specimen reception, decontamination, anonymization, and testing

Upon designation in May 2020 as a COVID-19 testing centre, the BSL immediately embarked on developing standard operating procedures to guide specimen reception, decontamination of primary and secondary sample containers, anonymisation, risk mitigation, testing and quality assurance procedures, including algorithms of test result interpretation, re-testing of all inconclusive results and reporting of validated results to the MOH. An important part of the standard operating procedure was daily decontamination of workspaces with 5% Chemgene (Medimark Scientific Limited, Kent, England), before and after use and in case of spillage. Surfaces decontaminated included workbenches, thermocyclers, nucleic acid extraction robots, centrifuges and vortexes), pipettes, bio-safety cabinets and door handles. To track down potential contamination, all surfaces listed above were swabbed every Monday with a disposable sampler (Qingdao Yongqiang Huashang Medical Technology Co., Ltd., Danyang, China) and tested for SARS-CoV-2. All surfaces had to have a negative test before nasal sample testing commenced. This procedure was repeated whenever contamination was identified or suspected.

Semi-automated extractions of RNA from nasal and oropharyngeal samples were performed using either the KingFisher Flex (Thermo Fisher Scientific Inc., Waltham, MA, United States) that can perform 96 extractions per hour and or MagPurix Evo® 24 (Zinexts Life Science, Taipei, Taiwan) that performs 24 extractions per hour. We also braced for manual extractions, in case the semi-automated kits ran out. For polymerase chain reaction (PCR) testing, the laboratory had the ABI 7300 and 7500 systems (Applied Biosystems, Foster City, California, United States) that can perform 96 tests per 2-h run and the Magnetic Induction Cycler (Bio Molecular Systems, Queensland, Australia) that performs 48 tests in a 2-h run. Samples came from different parts of the country and were transported in cool boxes. Our estimated maximum output per 24 h was 750 tests. Supplies for RNA extractions and testing were provided by the MOH. Because of global shortages of sequencing reagents, the sequencing efforts at the BSL was supported by the United States Department of Defense Global Emerging Infectious Surveillance programme. All kits (extractions and PCR) were validated against known positive samples before use. For quality assurance, external quality assessment samples were provided by the KEMRI (Nairobi, Kenya), National Public Health Laboratory (Nairobi, Kenya), One World Accuracy (Vancouver, Canada) and Thistle, Johannesburg (South Africa).

Strict procedures were implemented for entry into the laboratory and weekly testing of personnel for SARS-CoV-2 was required. Entry to the laboratory required a COVID-19 wellness self-assessment. Personnel were asked not to report to work if they had any of the following symptoms: fever ≥ 37.8 °C, sore throat, flu-like symptoms, direct contact or taking care of a COVID-19 patient, direct or accidental unprotected contact with samples suspected to contain SARS-CoV-2. To encourage compliance, absence of work on suspicion of having contracted COVID-19 was considered administrative leave and was not deducted from employees’ leave days.

## Lessons learnt

### Strict personnel procedures prevented disease spread in the laboratory

Over the 21-month testing period (May 2020 to January 2022), seven out of 11 laboratory personnel tested positive for COVID-19. These mitigating interventions were considered successful and considering the slow spread of the infection over the testing period, it is unlikely that the infections originated from inside the BSL.

### Initial travel restrictions and lockdowns slowed virus spread, but impacted SARS-CoV-2 supplies

**FIGURE 3:**
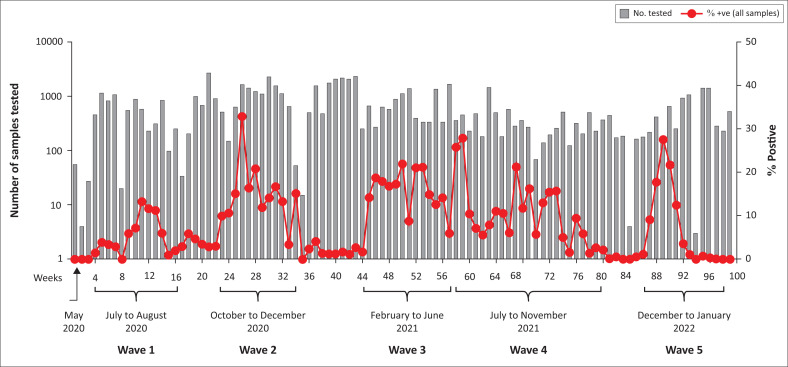
Five waves of COVID-19 in the samples tested at the Basic Science Laboratory, Kenya Medical Research Institute/Walter Reed Project, Kisumu Field Station, May 2020 – January 2022.

Because initial sample accrual was based on active case detection in each county, zero samples were brought to the laboratory in March 2020 or April 2020. In the 4th week of May 2020, we received a batch of 452 respiratory samples collected at the port of Busia from truck drivers. Of the 452, only seven (1.6%) were positive for SARS-CoV-2. From June 2020 onwards, the sample sources diversified to include the community, truck drivers, hospitals and military personnel. From May 2020 to January 2022, five waves of COVID-19 were discernible (Figure 3). The first wave started picking up steam in the first week of June 2020 at 3.0%, reached peak level by mid-July 2020 at 13.2% and thereafter declined steadily, remaining at 3.0% till end of September 2020. The second wave was discernable from the first week of October 2020 at 9.0%, reached peak level of 32.8% by end of that month, and thereafter declined steadily to 1.0% by the end of January 2021. Probably fueled by the Christmas and New Year festivities, wave three emerged suddenly in Nairobi and its environs from the second week of February 2021 at 14.2%. Although the wave had burned out in Nairobi by June 2021, it was still going on in western Kenya. Wave four emerged while wave three was still in progress. Therefore, there was no clear separation between these two waves and by the time wave four started in July 2021, infection rates from wave three were above 5%. Wave four did not die off until November 2021, making it the longest wave in Kenya. Unlike wave four, wave five emerged stealthily in December 2021, spread quickly, and died off as quickly as it had emerged by January 2022, making it the shortest COVID-19 wave in Kenya. Since then, infection rates have been minimal, forcing the MOH to reconsider the mandatory requirement for masking.

### Waves caused by variants of concern had a higher viral load

**FIGURE 4:**
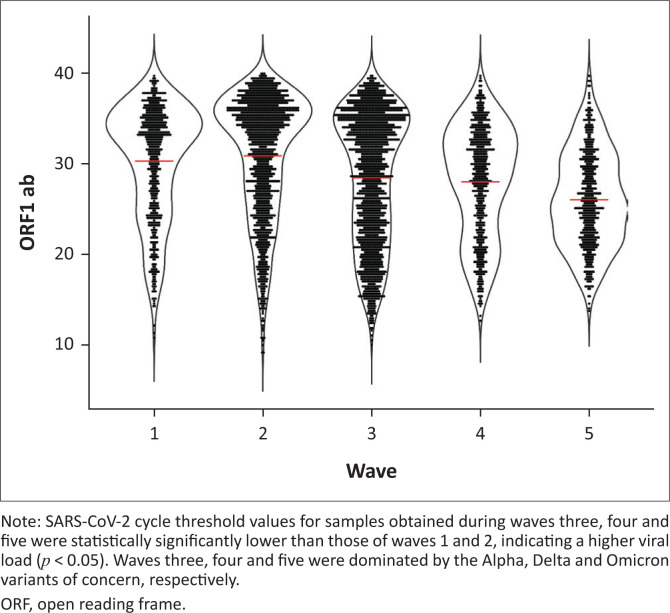
Scatter plot showing the viral load as determined from cycle threshold values for the five waves tested at the Basic Science Laboratory, Kenya Medical Research Institute/Walter Reed Project, Kisumu Field Station, May 2020 – January 2022.

Real-time PCR cycle threshold (Ct) values for the SARS-CoV-2 ORF1ab gene in samples obtained during the five waves were used as surrogates for tracking viral load (Figure 4). Samples tested during the third, fourth and fifth waves had significantly higher lower mean Ct values (28.439 *±* 0.304, 28.013 ± 0.251 and 26.033 ± 0.225 standard error of the mean, respectively) compared to wave one (30.303 ± 0.256 standard error of the mean) and wave two (30.890 ± 0.296 standard error of the mean, *p* < 0.05), indicating a higher viral load. From our sequence data, wave three was dominated by the Alpha variant of concern (originally identified in the United Kingdom), four by Delta (originally identified in India) and five by Omicron (originally identified in South Africa).^4^ These variants of concern have been associated with higher viral loads and/or doubling time.^5,6,7^ We recognise that there are caveats in relying on Ct values, but as suggested by Hay et al., at a population level, Ct values can be used to explain the trajectory of the COVID-19 epidemic.^8^

### Increased test demand put a huge strain on testing reagents and supplies

The automated extraction kits were the first to be depleted, and resupply was erratic due to banning of flights in and out of the country, coupled with increased global demand and competition. In addition, because infection rates in Kenya, and indeed in Africa, were still very low compared to global rates, Kenya was at the bottom of the ladder on supply priorities. The laboratory resorted to manual extraction using kits supplied by MOH, philanthropists and different embassies. Because of the increased testing demands and to save on resources and time, a sample pooling strategy was adopted. This was done when the infection rates were not more than 5%.

### Creation of process flow units reduced errors and increased efficiency

**FIGURE 5:**
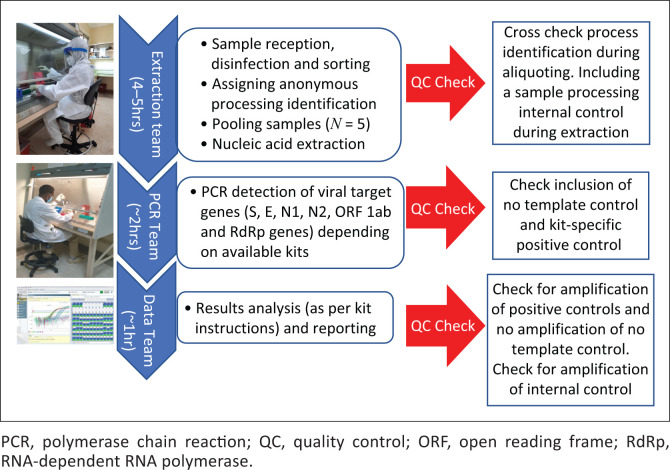
Laboratory personnel work units consisting of extraction, PCR, and data with quality control checks at various stages of sample processing at the Basic Science Laboratory, Kenya Medical Research Institute/Walter Reed Project, Kisumu Field Station, May 2020 – January 2022.

At the height of testing, the laboratory was receiving over 500 respiratory samples per day. Laboratory personnel organised themselves into work units consisting of *Extraction, PCR, and Data with Quality Control (QC) checks* at various stages (Figure 5). The extraction team was located in the ‘dirty room’ and donned complete body suits and facemasks. They were responsible for sample receipt and disinfection, sorting and assigning processing IDs, sample pooling and the nucleic acid extractions. Work units helped in two main areas. First, they reduced error rates, because teammates could QC each other. Second, because of increased workload that required working late and during weekends, shifts were easier to organise without too much disruption of sample processing and analysis.

The PCR team worked in the ‘clean room’ equipped with a level 2 biosafety cabinet where PCR master mixes and extracted nucleic acids were added before being loaded in thermocyclers that were located in a separate room. This separation further reduced the chances of amplicon cross contamination. Three personnel were designated to perform the PCR, with at least two working together at any given time. The PCR teams anticipated completion of RNA extractions to allow the PCR process to commence immediately. Real-time PCR amplification took 2 h. Once complete, amplification output was analysed, quality assurance checked and interpreted. Samples in the negative pools were reported as negative. Samples in the positive pools were re-extracted individually and re-tested. Each real-time PCR reaction included the human RNase P gene (RNase P) to check for sample integrity. A total of 64 643 samples were tested in the reporting period, of which 5376 (8.3%) were positive for SARS-CoV-2. Resampling was requested in 668 (1.4%) samples because of poor sample quality. Of the 5376 positive samples, 1034 with Ct values ≤ 33 were sequenced on an Illumina MiSeq (Illumina, San Diego, California, United States).

## Recommendations

A great source of pride for any laboratory contemplating SARS-CoV-2 testing in support of the COVID-19 pandemic control is the realisation that the effort is part of what the World Health Organization refers to as critical preparedness, readiness and response actions that save lives.^9^ A fundamental lesson to embrace is that there is no ‘one-size-fits-all’ approach, and that adaptation of traditional workflows and processes are crucial. Given the COVID-19 diagnostic demand amidst the global shortfall in supplies, the BSL would not have been able to meet the testing requests without adopting the specimen pooling strategy. With these realisations, the BSL was able to quickly adapt to increased testing demand dictated by an emergent new disease. Of the 323 272 COVID-19 confirmed cases in Kenya, BSL contributed 5376 (1.7%) positive samples. The nasopharyngeal swab collection centres did a commendable job of ensuring sample integrity, despite the long distance to the testing laboratory.

## References

[1] Ministry of Health. First case of coronavirus disease confirmed in Kenya [homepage on the Internet]. 2020 [cited 13 March 2020]. Available from: https://www.health.go.ke/first-case-of-coronavirus-disease-confirmed-in-kenya/

[2] Hasell J, Mathieu E, Beltekian D, et al. A cross-country database of COVID-19 testing. Sci Data. 2020;7:345. 10.1038/s41597-020-00688-8 33033256 PMC7545176

[3] Bugembe D, Kayiwa J, Phan M, et al. Main routes of entry and genomic diversity of SARS-CoV-2, Uganda. Emerg Infect Dis. 2020;26(10):2411–2415. 10.3201/eid2610.202575 32614767 PMC7510740

[4] WHO. Tracking SARS-CoV-2 [homepage on the Internet]. 2022 [cited 2022 June]. Available from: https://www.who.int/activities/tracking-SARS-CoV-2-variants?msclkid=c2729b29d10811eca952b5e97b4da00a

[5] Kidd M, Richter A, Best A, et al. S-variant SARS-CoV-2 lineage B1.1.7 is associated with significantly higher viral loads in samples tested by Thermo Fisher TaqPath RT-qPCR. J Infect Dis. 2021;223(10):1666–1670. 10.1093/infdis/jiab082 33580259 PMC7928763

[6] Hill KJ, Dewar R, Templeton K. A multiregional evaluation of Ct values in SARS-CoV-2 VOC-20DEC-01 variant. J Infect Dis. 2021;224(5):927–928. 10.1093/infdis/jiab303 34467990

[7] Karim SSA, Karim QA. Omicron SARS-CoV-2 variant: A new chapter in the COVID-19 pandemic. Lancet. 2021;398(10317):2126–2128. 10.1016/S0140-6736(21)02758-6 34871545 PMC8640673

[8] Hay JA, Kennedy-Shaffer L, Kanjilal S, et al. Estimating epidemiologic dynamics from cross-sectional viral load distributions. Sci. 2021;373(6552):eabh0635. 10.1126/science.abh0635 PMC8527857 34083451

[9] Critical preparedness, readiness and response actions for COVID-19 [homepage on the Internet]. World Health Organization; 2021 [cited 27 May 2021]. Available from: https://www.who.int/emergencies/diseases/novel-coronavirus-2019/technical-guidance/critical-preparedness-readiness-and-response-actions-for-covid-19

